# The Effects of Malting and Extrusion on the Functional and Physical Properties of Extrudates from Malted Brown Rice and Pigeon Pea Flour Blends

**DOI:** 10.3390/foods14030422

**Published:** 2025-01-28

**Authors:** Chinenye Azuka, Amarachi Onwuchekwa, Adaora Nwosu, Mel Holmes, Christine Boesch, Gabriel Okafor

**Affiliations:** 1 School of Food Science and Nutrition, University of Leeds, Leeds LS2 9JT, UK; m.j.holmes1@leeds.ac.uk (M.H.); c.bosch@leeds.ac.uk (C.B.); 2 Department of Food Science and Technology, University of Nigeria, Nsukka 410001, Nigeria; amarachi.onwuchekwa@unn.edu.ng (A.O.); adaora.nwosu@unn.edu.ng (A.N.); gabriel.okafor@unn.edu.ng (G.O.)

**Keywords:** response surface methodology, extrudates, expansion index, internal structure, rheology

## Abstract

Malted grains subjected to extrusion technology could have better quality indices than non-malted grains. The effects of malting and extrusion on the functional and physical qualities of foods extruded from malted brown rice and pigeon pea flour blends were investigated. Malted pigeon pea and brown rice flours were processed into blends, extruded under various conditions of feed moisture levels (15–20), feed compositions (8–30%), and barrel temperatures (100–130 °C), and analyzed using Response Surface Methodology with a Box–Behnken design. The impacts of malting and extrusion were assessed on the following functional qualities: bulk density, rheology, swelling capacity, water absorption capacity, and solubility. The physical quality assessment included a 2-D photographic representation of the extrudates, a microscopic assessment of their internal structure, expansion index, color parameters (L*, a*, b*), and alterations in the color index. Increased feed moisture, malted pigeon pea, and decreased barrel temperature resulted in a higher bulk density (0.72 to 0.84 g/cm^3^) of the extrudates. There was a decrease in water absorption capacity (6.82–4.49%) with an increase in barrel temperature above 100 °C. All the samples showed a decrease in viscosity with increasing shear rate. At low barrel temperatures, feed compositions, and feed moistures, extrusion led to increases in the expansion index (3.5 to 12.94) and the color lightness (66.83–81.71) of the extrudates. Samples with a higher proportion of malted brown rice showed a higher expansion index, lower bulk density, and lighter color of the extrudates.

## 1. Introduction

The ever-growing population demands that food security be achieved by developing sufficient food for individuals from all walks of life, regardless of health conditions. Subjecting underutilized grains to processing technologies to improve their nutritional value and acceptability is a means of ensuring food security. Malting is an inexpensive and complex metabolic process that improves the palatability and digestibility of food, as well as the availability of secondary metabolites and bound nutrients. During malting, the lipids, carbohydrates, and storage proteins in the seed are broken down to supply the energy and amino acids needed for the growth of a new sprout and for subsequent plant development [1,2,3]. Enzyme inhibitors are also degraded during malting, thereby making foods more accessible to intestinal enzymes to improve digestion [2]. These benefits have been evidenced in cereals [2,4,5,6,7] and pulses [8,9,10,11] subjected to malting, which has ultimately led to improved nutrition and to the support of goal 3 of the sustainable development goals (SDGs).

Brown rice, a whole-grain cereal, has shown reduced phytates and a higher content of dietary fiber, B vitamins, tocopherols, minerals, gamma-aminobutyric acid (GABA), ferulic acid, and γ-oryzanol when subjected to malting in comparison with white rice, non-malted brown rice, and parboiled milled rice [1,12]. Pigeon pea, scientifically known as *Cajanus cajan*, is considered the most underutilized pulse globally because of its challenging cooking process, as it requires a significant amount of time to soften when heated. When malted, it exhibits more secondary metabolites (phenolic compounds and flavonoids) than its non-malted counterpart [13]. Pigeon pea shows great potential, as it is the only pulse crop that can still produce grains in conditions under which other pulse crops and cereals are affected by wilt and dryness caused by moisture stress [14]. It has a relatively high protein content (24–40%) compared to soybean (35%), cowpea (19–27%, bambara groundnut (17–25%), and African yam bean (19.5–29.5%) [15].

Obtaining an optimum nutritional content of malted foods depends to a great degree on the processing method used in preparing the foods for consumption, as some types of cooking and processing methods increase the loss of nutrients [16]. Extrusion cooking is a processing technology that uses high temperature and short processing times to create various products from diverse food components by applying variable shear stress and mass flow in segmented screw and barrel sections [17]. It has been shown that most nutrients are preserved when exposed to extrusion cooking compared to conventional cooking methods like boiling, frying, and roasting [16,18,19]. In extrusion cooking, the macro molecules of agricultural materials are modified with different combinations of process parameters to prepare novel products such as breakfast cereals [20,21], savory snacks [22], pre-cooked flours [23,24], cereal-based baby food [25], and textured proteins [26,27]. Extruded products exhibit different properties than their non-extruded counterparts in terms of their nutritional, functional, physical, and acceptability levels.

The functional properties (bulk density, rheology, swelling capacity, water absorption capacity, and solubility) of extruded products are critical for the techno-functionality of the extrudates. The physical properties of extruded products are equally critical quality indicators for the overall acceptability of the extrudates. The physical properties that express the effectiveness of extrusion are appearance, expansion index, specific density, particle size, mass flow rate, specific volume, color parameters (L*, a*, b*), and hardness. Whilst there is extensive research on the nutritional and physicochemical properties of extruded non-malted cereals and pulse blends, there is a paucity of information on the physical properties (the first determinant of acceptability) and functional properties of malted blends, with an expected higher nutritional value.

Whilst the impact of extrusion on the physical qualities of non-malted brown rice and pigeon pea blends has been investigated [28], there is a lack of knowledge on the impact of malting on the functional and physical characteristics of extrudates from blends of malted brown rice and pigeon pea flours. This study assessed how malting and extrusion conditions impact the functional and physical properties of extruded of malted brown rice and pigeon pea flour blends. The parameters assessed were bulk density, rheology, swelling capacity, water absorption capacity, and solubility, while the physical qualities assessed included a 2-D photographical representation of extrudates as they emerged from the die, a microscopic assessment of their internal structure, the expansion index, color parameters (L*, a*, b*), and alterations in the color index.

## 2. Materials and Methods

### 2.1. Acquisition of Raw Materials

The brown rice (FARO 44 variety) [100 kg] *Oryza sativa glaberrima* was obtained during the 2020 harvest season from the National Cereal Research Institute in Badeggi Bida, Niger State, Nigeria. The Red Pigeon pea (*Cajanus cajan* L.) [50 kg] was obtained at Ogige market in Nsukka LGA in Enugu State, Nigeria. The raw materials underwent cleaning by winnowing and sorting to remove impurities and were then packaged in Ziploc bags. They were subsequently stored at room temperature (30 °C) until they were needed.

### 2.2. Malting of Brown Rice and Pigeon Pea

The malting of brown rice was carried out according to the method of Patil and Khan [29] with modifications. After the initial cleaning to remove extraneous materials, the brown rice (3 kg) was soaked in 1:2 water at 28–35 °C for up to 12 h, with water changes every 4 h. After 12 h, the water was drained, and grains kept in moist condition on a malting bag for 20–24 h until a 0.5–1 mm long sprout from the germinating brown rice grains were observed. The grains were thereafter harvested and oven-dried to constant weight in a convection air oven (Geotechnical multi-purpose laboratory oven, Geotechnical equipment, Milton Keynes, UK) at 50 °C to avoid gelatinization until brittle. The rootlets were removed by abrasion and winnowing and the grain milled in a hammer mill (Lerford Machine LLC, Lakewood, CO, USA) repeatedly to prepare malted brown rice flour. The flour obtained was sieved using a sieve with a mesh size of 0.5 mm and the samples stored at −20 °C in Ziploc bags in a moisture-free environment until required. This process was repeated until 40 kg of germinated brown rice was obtained.

The malting of pigeon pea was carried out according to the method of Uchegbu and Ishiwu [13] with modifications. Pigeon pea (1.5 kg) was washed thoroughly using tap water and soaked in 1:5 water of 28–35 °C for 15 h. The water was changed every 4 h to avoid fermentation. The water was drained, and the seed was placed in a malting bag to germinate for 56 h at 28 °C in darkness. The germinated seeds were oven-dried at 50 °C for 12 h or until they were friable, and then the rootlets were detached. The dried sprouted seeds were milled in a hammer mill (Lerford Machine LLC, Lakewood, CO, USA) repeatedly, sieved using a sieve with a mesh size of 0.5 mm into flour, and thereafter packed in Ziploc bag and stored at −20 °C until needed. This process was repeated until 30 kg of germinated pigeon pea was obtained.

### 2.3. Designing Formulations Using Response Surface Methodology

Minitab software was utilized to establish a Response Surface Methodology (RSM) in a three-factor Box–Behnken design. The operating ranges and three standardized levels were determined following multiple preliminary trials and operating ranges shown in Table 1. Feed moisture content: 15–20 (*X*_1_), barrel temperature 100–130 °C, (*X*_2_) and feed composition 8–30% (amount of pulse combined with malted and un-malted rice flour—*X*_3_), were the independent variables considered (Table 2). The feed moisture content (*X*_1_) for each design point was obtained using the formula denoted as (Equation (1)).

(1) $$Y=\frac{\left(M_{f}-M_{i}\right)}{100-M_{f}}\times S_{w}$$

where *Y* = amount of water to be added (mL); *M_f_* = final moisture content; *M_i_* = initial moisture content; *S_w_* = sample weight (g).

The functional and physical attributes of the extrudates (*Y*) were taken as the dependent variables of the design experiments; all other parameters were kept constant. Regression analysis was utilized to assess the relationship between the dependent and independent variables and to produce regression equations. The response was represented by a second-order polynomial equation, denoted as Equation (2).

(2) $$Y=\beta_{0}+\beta_{1}X_{1}+\beta_{2}X_{2}+\beta_{3}X_{3}+\beta_{11}X_{1}^{2}+\beta_{22}X_{2}^{2}+\beta_{33}X_{3}^{2}+\beta_{12}X_{1}X_{2}+\beta_{13}X_{1}X_{3}+\beta_{23}X_{2}X_{3}+\varepsilon$$

### 2.4. Determination of Physical Properties 

#### 2.4.1. Photographic (Longitudinal Section) Image of the Extrudates

The appearance of the extrudates was captured in the presence of two fluorescent light tubes hung over the extrudates as described by [30]. The extrudates were laid on a cleaned, smooth, black surface. The image of the neatly laid extrudates was captured using the 48 mega-pixel camera of an iPhone 14 pro max at an equal distance of 8–10 m. Precaution was taken to ensure there were no blurred lines, and the images were further edited.

#### 2.4.2. Development of 2-D Slice Microscopic Expanded Product

The microscopic internal attributes of the extruded samples were viewed under a microscope (ZEISS LSM 980, Jena, Germany). Segments (2-D) of all the samples, approximately 1.0 mm thick, were cross-sectioned with a razor, with each sample placed on a transparent slide and viewed under the microscope. The distance between the microscope and samples was adjusted to 100× magnification (1 µm). The internal structure of samples with larger expanded surfaces was viewed repetitively from the sides to capture the porous structure. The perfect typical representation of the internal structure of each extrudate was reported.

#### 2.4.3. Sectional Expansion Index

The sectional expansion index (*SEI*) of the extrudate was determined by dividing the cross-sectional areas of the extrudate (De) by the cross-sectional areas of the die opening (Dd) using the method described by Patil et al. [31] and expressed as Equation (3). The diameter of the extrudates was determined, as well as the die opening and the area, calculated as in Equation (3). Ten (10) measurements were taken for each sample

(3) $$SEI={\left(\frac{\mathrm{De}}{\mathrm{Dd}}\right)}^{2\;}$$

#### 2.4.4. Determination of Color Characteristics 

The colors of the extruded foods were analyzed using a Minolta Color Reader (CR-10, Minolta Co. Ltd., Tokyo, Japan) based on the L^+^, a*, and b* system. The equipment was standardized with a white square, which had standards of L = 97.01, a = +0.13, and b = −0.58. The L^+^ value represents the lightness of the product, from black (0) to absolute white (100); the a* value indicates the red-to-green color range (a+ = 0–60 for red and a− = 0–(−60) for green); and the b* value represents the blue-to-yellow color range (b+ = 0–60) for yellow and b* = 0–(−60) for blue. Ten (10) measurements were taken for each sample, as described by Danbaba [32].

The whiteness index and change in color is expressed in Equations (4) and (5), as reported by Patil et al. [31]

(4) $$\mathrm{The}\;\mathrm{whiteness}\;\mathrm{index}=W={\left\{{\left(100-L\right)}^{2}+a^{2}+b^{2}\right\}}^{0.5}$$

(5) $$\mathrm{Change}\;\mathrm{in}\;\mathrm{color}\;={\left\{\left(△a{)}^{2}+(△b^{2}\right)+\left(△L^{2}\right)\right\}}^{0.5}$$

### 2.5. Determination of Functional Properties of the Extrudates

#### 2.5.1. Bulk Density (BD)

Bulk density (BD) was measured following the procedure outlined by Banki et al. [28] with minor adjustments. Five grams of the extruded sample was placed into a clean 10 mL measuring cylinder and tapped until it no longer showed a decrease in volume. The measurements were conducted three times, and the average volume was determined as the packed volume. The weight of the extruded sample per unit volume was determined as the bulk density (BD) using a standard formula (Equation (6)):

(6) $$\mathrm{Bulk}\;\mathrm{density}\;\left(\frac{g}{\mathrm{mL}}\right)=\frac{\mathrm{weight}\;\mathrm{of}\;\mathrm{extruded}\;\mathrm{sample}\left(g\right)}{\mathrm{volume}\;\mathrm{occupied}\;\mathrm{by}\;\mathrm{the}\;\mathrm{extruded}\;\mathrm{sample}\left(\mathrm{mL}\right)}$$

#### 2.5.2. Viscosity Measurement of the Extrudates

Viscosity measurement was conducted using a rheometer (Kinexus Rheometer, Malvern Instruments, Malvern, UK) which was first calibrated using water. The extrudates were selected based on their differences in the extrusion factors of feed moisture, barrel temperature, and feed composition. The extrudate flour was dispersed in water to 25% concentration (comparable thickness for consumption) at a temperature of 25 °C. Care was taken to ensure the paste was homogeneous and bubble-free. A small geometry (<25 mm) was selected, which is suited to highly viscous (>10 Pa·s) samples. A small amount of sample (to avoid spillage when plate is clamped) was applied onto the lower measuring surface and the rotational plate clamped. The measurement mode and shear rate were selected and the measurement started. The rheometer applied controlled stress to the sample and measured the resulting force as well as the viscosity. The measurement data were recorded in real-time.

#### 2.5.3. Swelling Capacity

The swelling capacity was determined by the method described by Okaka and Potter [33] with major modifications. A 100 mL graduated cylinder was filled with the sample to 10 mL mark. The distilled water was added to give a total volume of 50 mL. The solution was stirred left to stand for 2 min. After 2 min, the solution was stirred and allowed to stand for a further 8 min. The volume occupied by the sample was taken after the 8th min. Swelling capacity was calculated as shown in Equation (7):

(7) $$\mathrm{Swelling}\;\mathrm{capacity}\left(\%\right)=\frac{\mathrm{final}\;\mathrm{volume}-\mathrm{initial}\;\mathrm{volume}}{\mathrm{initial}\;\mathrm{weight}}\times \frac{100}{1}$$

#### 2.5.4. Water Absorption Capacity (WAC) and Solubility Index

Water absorption capacity and solubility index values were determined according to the modified method of Gandhi and Srivastava [34] and Yousf et al. [35], respectively; one gram of sample was mixed with 10 mL distilled water in centrifuge tubes and then allowed to stand for 30 min. The sample was centrifuged at 4000 rpm for 15 min. The supernatant was poured into a Petri dish and dried at 110 °C while the tube with sediment was weighed. Water absorption capacity (grams of water per gram sample) and solubility index values were calculated using the formula Equations (8) and (9), respectively.

(8) $$\mathrm{WAC}=\frac{W_{2}-W_{1}}{W_{0}}$$

(9) $$\mathrm{Solubility}=\frac{Wt\;of\;dried\;supernatant}{\mathrm{Weight}\;\mathrm{of}\;\mathrm{dry}\;\mathrm{solid}}$$

where *W*_0_ = weight of dry sample (g); *W*_1_ = weight of tube and dry sample (g); *W*_2_ = weight of tube plus sediment (g).

#### 2.5.5. Optimization of the Responses

This study was designed to produce extrudates from flour blends of malted brown rice–pigeon pea formulations with high expansion index, water swelling and absorption capacity, solubility, lightness, b* index; and low bulk density, a* index, and moisture. MINITAB’s Response Optimizer was adopted for the simultaneous numerical optimization of the multiple responses, to search for a combination of independent variable levels that simultaneously satisfied the target requirement placed on each response and factors. Numerical optimization require that goals (none, maximum, minimum, target or range) should be set for the variables and response, where all goals are combined into one desirable function [36,37]. In this study, the acceptable set of conditions that met all the goals, the independent variables, were a feed moisture content level of (15–20), barrel temperature (100–130 °C), and feed blend composition (8–30 g/100 g), which were all set within range, while expansion index, b*, lightness, water absorption capacity, water swelling capacity, and solubility, were set at 5, with a* set at 1. Gupta et al. [37] reported that the ‘importance’ score of a goal is within 1 to 5, and setting goal importance at 3 indicates that the variable is considered to be equally important. However, Anuar et al. [36] reported that when it is set at 5, the objective is to obtain a response at maximum level, which was applied in this study. Overall desirability (OD), which measures how well the set condition satisfies the combined goals for all the responses, was set between 0 and 1, where 1 represents ideal conditions and 0 indicates that one or more of the responses were outside the acceptability limits.

### 2.6. Statistical Analysis

The measurements were carried out in triplicate and reported as the mean value ± standard deviation. An ANOVA was conducted using Minitab software (version 17), with significance set at *p* < 0.05. The results from the Box–Behnken design were analyzed to assess the impact of feed moisture level, feed blend composition, and barrel temperature on the functional and physical properties, as well as the impact of malting. The model’s fitness was assessed using the coefficient of determination (R^2^), lack of fit, and *p*-values derived from the regression analysis.

## 3. Results and Discussion

### 3.1. Effects of Malting and Extrusion Variables on the Physical Properties of Rice–Pigeon Pea Extrudates

#### 3.1.1. Appearance

**Figure 1:**
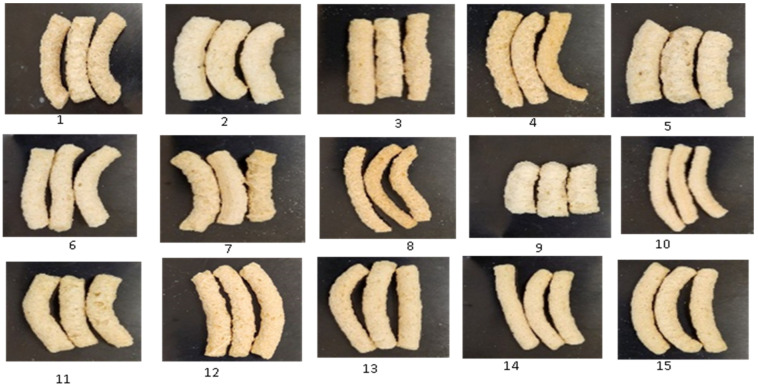
Two-dimensional photographic images of the physical state of malted brown rice–pigeon pea blend extrudates as affected by different processing variables. Key (*X*_1_, *X*_2_, *X*_3_) (*X*_1_ = feed moisture, *X*_2_ = extruder barrel temperature, *X*_3_ = feed composition): 1 (15, 100, 19); 2 (20, 100, 19); 3 (15, 130, 19); 4 (20, 130, 19); 5 (15, 115, 8); 6 (20, 115, 8); 7 (15, 115, 30); 8 ((20, 115, 30); 9 (17.5, 100, 8); 10 (17.5, 130, 8); 11 (17.5, 100, 30); 12 (17.5, 130, 30); 13 (17.5, 115, 19); 14 (17.5, 115, 19); 15 (17.5, 115, 19).

Figure 1 displays the images of extrudates as they exited the die under steady-state conditions for each experimental setting. The extrudates differed in physical appearance in terms of variations in expansion levels, surface roughness (smooth and corrugated surfaces), size, and color. This signifies variation caused by disparities in extrusion conditions and malting. Patil et al. [31] reported that the surface texture of the extrudate is a crucial physical quality for ready-to-eat snack products. It is greatly influenced by the raw material composition, the moisture level of the mix, and barrel temperature of the extruder. Those with lower moisture content (15%), less pigeon pea composition (8 g/100 g), and extruded at 100 °C, exhibited larger size and a rougher surface compared to those extruded at higher moisture contents (17.5–20%), higher barrel temperature (115–130 °C), and feed composition (19–30%). The strands of the latter were thinner and smoother. This observation aligns with the result of Danbaba et al. [38], who processed full-fat soybean flour with broken rice fractions. They noticed that when the moisture level reached 24%, the strands grew thinner and had a smoother surface. Conversely, at lower moisture content (8%) and when extruded at temperatures of 100 °C, the strands somewhat expanded in size and had a rougher surface. Nwabueze [39] noted that thin–smooth to thin–fine–smooth extrudates were produced when African breadfruits were mixed with a higher percentage of soybean and yellow maize and extruded with a greater moisture content.

**Figure 2:**
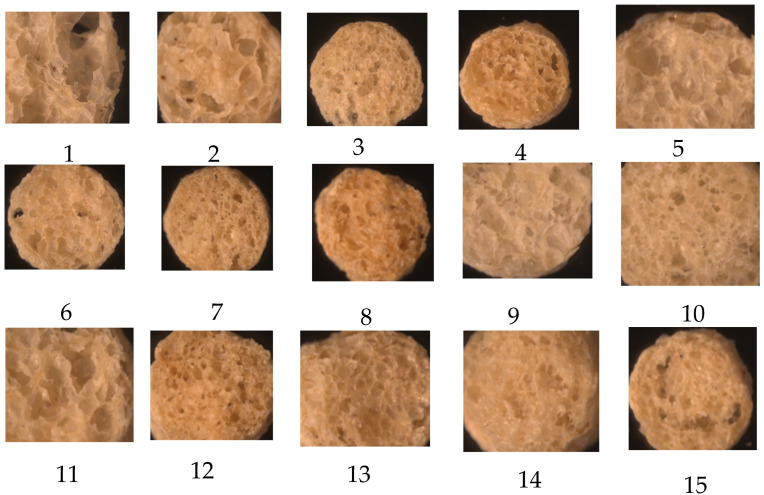
Microscopic view of internal structure of selected extrudates of each design point (magnification 100×). 1 (15, 100, 19); 2 (20, 100, 19); 3 (15, 130, 19); 4 (20, 130, 19); 5 (15, 115, 8); 6 (20, 115, 8); 7 (15, 115, 30); 8 ((20, 115, 30); 9 (17.5, 100, 8); 10 (17.5, 130, 8); 11 (17.5, 100, 30); 12 (17.5, 130, 30); 13 (17.5, 115, 19); 14 (17.5, 115, 19); 15 (17.5, 115, 19).

Malting grains decrease their molecular mass, leading to lighter extrudates and higher cellulose content. Malting did not alter the properties of pigeon peas, as the pulses were able to support structures, even after being malted. A high level of malted pigeon pea resulted in the extrudates being more rigid due to the creation of complex matrices between starch and protein. This caused a decrease in expansion and an increase in smoothness as the amount of malted pigeon pea in the samples increased (Figure 1). Samples containing a higher proportion of malted brown rice, which is abundant in low molecular weight compounds and non-starchy carbohydrates such as cellulose and fiber, showed increased puffiness and corrugated surfaces. This was attributed to the absence of large molecules and decreased pea content that affects the structure. Extrudates containing a significant amount of malted brown rice were lightweight and easily shattered because of decreased toughness. The materials exhibited loose patterns when observed under an optical microscope (Figure 2).

#### 3.1.2. Expansion Index (EI)

Table 3 displays the average impacts of feed moisture content, barrel temperature, feed material composition, and malting on the EI from the flour blends of extruded malted rice–pigeon pea. The expansion index (EI) increased from 3.5 to 12.94 [40] when the initial feed moisture level decreased from 20 to 15. While maintaining an intermediate barrel temperature of 115 °C, pigeon pea content decreased from 30% to 8%. Hagenimana et al. [41] and Danbaba et al. [38] reported that higher EI was noted with decreased moisture content from 22% to 16% and from 25% to 20%, respectively. Increased moisture content decreases the material’s shear strength and energy input, leading to reduced moisture evaporation at the die exit and, hence, hindering expansion. An optimal product expansion requires a specific narrow range of moisture. Additionally, there was a decrease in expansion index when the percentage of pigeon pea flour increased from 8% to 30%. In individual experiments, Danbaba [38], Yu et al. [42], and Devi et al. [43] all observed that the expansion ratio decreased when protein was added. Similarly, in this study, an increase in pigeon pea, a protein-rich pulse, led to a decrease in starch levels in the blend. This decrease may have hindered complete starch gelatinization, resulting in a lower expansion index. Higher starch–protein ratio results in the creation of a consistent starch matrix that impacts its expansion as it leaves the die [38,44]. A high expansion index is a favorable physical characteristic in puffed extruded meals, as it usually signifies a lighter and crispier product [38].

Malting had effect on the expansion index of the extrudates. Samples with a greater amount of germinated brown rice flour, without a higher protein mix, showed an expansion index ranging from 3.5 to 12.94. This was higher compared to the extrudates made from un-malted flour, which had an expansion index between 2.13 and 9.56, as reported by Banki et al. [28]. Increasing the incorporation of malted brown rice led to larger expansion of the extrudates, resulting in a highly porous interior structure with air voids. This may be due to the breakdown of the amylose–amylopectin structure, which increased the cellulose and cell-wall composites, resulting in a highly porous interior structure with air holes (Figure 2). Also, increasing the amount of malted pigeon pea, containing high amount of amino acids and protein, increased the starch–protein complex interaction, thereby hindering expansion.

**Figure 3:**
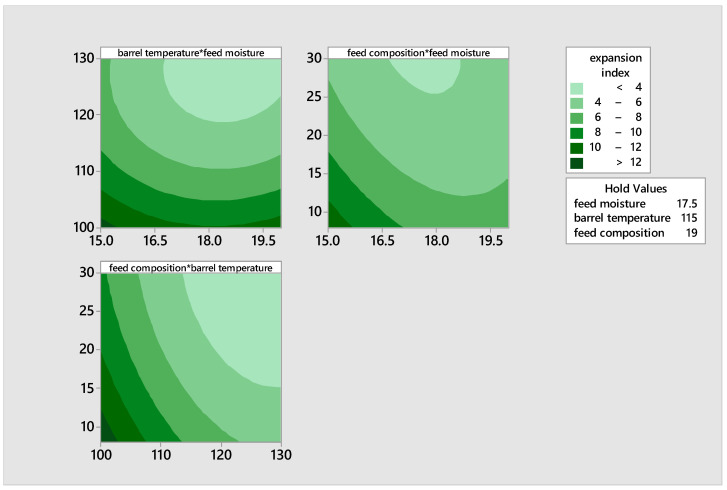
Contour plot illustrating the effects of moisture, feed composition, and temperature on the expansion index of the extrudates.

Figure 3 shows the effects of independent factors on expansion index by contour plot observations. At low temperatures and low feed moisture levels, the expansion index increases. This also occurs when there is an interaction between low feed composition and feed moisture. Furthermore, when the feed composition and the barrel temperature were low, the expansion index increased. The screw applies shearing force on starch and protein-based materials during extrusion, which, combined with temperatures at 100 °C, changes the material into a viscoelastic mass. Upon exiting the die, there is a sharp drop in pressure, causing the material to expand significantly (*p* < 0.05) [45]. The extrudate’s physical qualities, including density, expansion, and texture, are determined by the material composition (protein, starch, fiber, and moisture) and the extrusion process conditions [45].

The model fitness was evaluated using the coefficient of determination (R^2^), lack of fit, and *p*-values derived from the regression analysis. Table 4 displays the outcomes of the analysis of variance (ANOVA) for the quadratic models that were created. Equation (10) displays the regression equation for the expansion index expression.

(10) $$\begin{array}{cc} \mathrm{Expansion}\;\mathrm{index} & =239.8-9.44X_{1}-2.095X_{2}-1.396X_{3}+0.2634X_{1}X_{1}+0.008406X_{2}X_{2}\\ & +0.00614X_{3}X_{3}-0.00749X_{1}X_{2}+0.03301X_{1}X_{3}+0.00356X_{2}X_{3} \end{array}$$

The linear impact of the three parameters had a substantial influence on the expansion index of the blend at a significance level of *p* < 0.05. Equation (10) demonstrates that as each of the linear terms rises, there is a drop in the expansion index. The quadratic terms were statistically significant, with *p*-values of 0.000. The relationship between feed moisture, feed composition, barrel temperature, and their interaction was found to be statistically significant (*p* < 0.05). The *p*-values for these factors were all 0.00. The R^2^ and adjusted R^2^ values were determined to be 87.03% and 86.49%, respectively. This indicates that the model equation could explain 87.03% and 86.49% of the variations, respectively. The model was significant, as was the lack of fit. There was a coefficient of variation (CV) of 8.47% which indicates a reasonable amount of variance in the experiments. Nath and Chattopadhyay [46], concluded that models with a coefficient of variation (CV) exceeding 10% are not considered credible.

#### 3.1.3. Color Characteristics of the Extrudates

Table 3 showed the color attributes of the extrudates. The products exhibited a variety of lightness (L^+^) values, specifically ranging from 66.83 to 81.71. The lowest lightness value was obtained with a feed composition of 30%, a feed moisture of 20, and a barrel temperature of 115 °C. Conversely, the highest lightness tone was attained with a feed moisture of 17.5, a barrel temperature of 100 °C, and an incorporation of 8% pigeon pea. The decrease in lightness is likely due to the occurrence of the Maillard browning reaction and the caramelization of sugars in the feed composition. It could also be due to the degradation of pigments in the raw materials during processing. The lightness of the extrudates reduced as the integration of 8% malted pigeon pea flour (PGPF) increased to 30%, indicating a reduction in brightness. The two distinct L^+^ values (sample 8 and 9) were observed at temperatures of 100 °C and 130 °C, respectively, under varying moisture levels and pea inclusion conditions (Table 3). The findings were consistent with the research conducted by Danbaba et al. [38], which indicated that when the moisture level was below 20% and the extrusion temperature above 100 °C, the extruded starch underwent darkening mostly owing to prolonged residence time and higher viscosity, resulting in a lower L^+^ value. In a study conducted by Gutkoski and El-Dash [47], it was shown that in an extruded oat product, luminosity declined in a linear manner as the barrel temperature increased. The study used an initial moisture content of 17 to 24% and a barrel temperature ranging from 90 °C to 150 °C. The results aligned with the findings presented in Table 3, which suggested that the most significant decrease in luminosity occurred in the sample with a high level of pea incorporation and a higher temperature. This could be attributed to the intensity of browning actions such as the Maillard reaction, caramelization (due to increased presence of simple sugar from malted flours), as well as the potential degradation of pigments that occurs during heat treatments [38,48].

**Figure 4:**
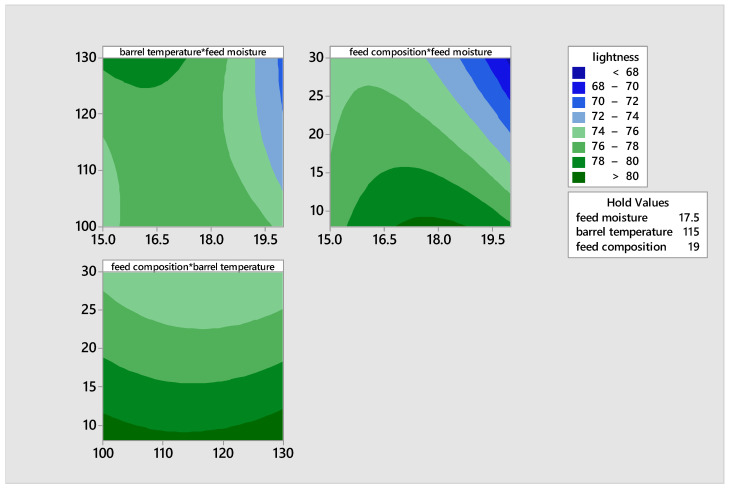
Contour plots illustrating the effects of moisture, feed composition, and temperature on the lightness of the extrudates.

According to Figure 4, the brightness of the product decreased when the feed composition, barrel temperature, and moisture content increased. An observation was made that the lightness reduced as the feed moisture and feed composition increased, whereas the lightness increased at low feed composition and barrel temperature. The contour plot provides a comprehensive and improved depiction of how variables impact the mixes, even when the relationship is not easily explained by a linear model. The disparities suggest the most appropriate point that would yield satisfactory quality in relation to this parameter.

(11) $$Lightness\;L^{+}=-117.5+22.07\;X_{1}-0.034X_{2}+1.281X_{3}-0.4432X_{1}X_{1}+0.00397X_{2}X_{2}+0.00273X_{3}X_{3}-0.0488X_{1}X_{2}-0.0842X_{1}X_{3\;}-0.00167X_{2\;}X_{3}\;$$

Table 4 displays the analysis of variance (ANOVA) for the quadratic model that was developed. The results indicate that the model is statistically significant, as evidenced by a *p*-value of 0.000, specifically in relation to the lightness of the extrudates. Equation (11) displays the regression equation for the variable of lightness. The moisture content and composition of the feed had a significant impact on the lightness of the extruded mix, with a *p*-value of less than 0.05. The quadratic effects of barrel temperature and feed composition were not statistically significant, as indicated by their respective *p*-values of 0.054 and 0.466. The interaction between feed moisture and barrel temperature was shown to be statistically significant, as well as the interaction between feed moisture and feed composition, with a significance level of *p* < 0.05. The R^2^ and adjusted R^2^ values were determined to be 86.53% and 83.06%, respectively. The coefficient of variation (CV) was 0.99%, indicating a good level of precision in the experiment.

#### 3.1.4. Responses of the a* Color Index

**Figure 5:**
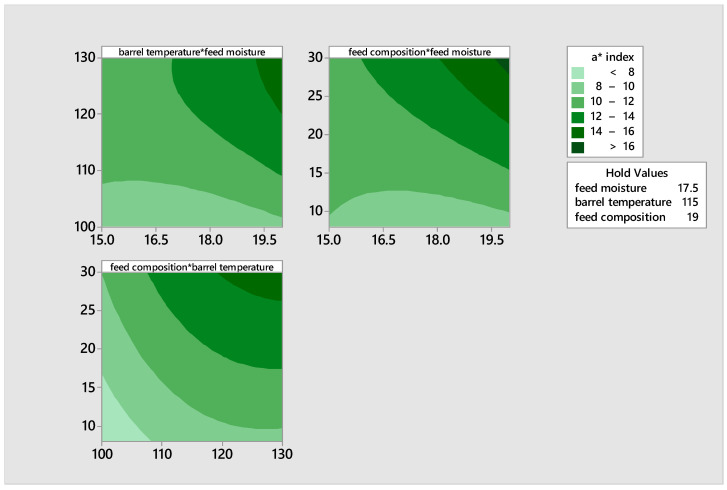
Contour plots illustrating the effects of moisture, feed composition, and temperature on the a* of the product.

The responses of the a* color index, which indicates the range from green to red on the chroma chart, exhibited variation based on the treatments. The a* index value of the extrudate varied between 6.95 and 17.48 (Table 3), showing that all samples exhibited a reddish brightness. The extrudate with the most intense red color was produced by extruding a mixture of feed moisture level of 20, a barrel temperature of 115 °C, and a feed composition of 30%. All of the extrudates displayed a positive red a* index chroma value, indicating an absence of greenness. The contour plot in Figure 5 showed that the intensity of redness increases as the barrel temperature, feed composition, and moisture increase. These factors are likely to have an impact on the acceptability of the product.

The quadratic model is statistically significant with a *p*-value of 0.0000. Equation (12) displays the regression equation for the variable a* index. The coefficients for the linear and quadratic terms of feed moisture and barrel temperature were statistically significant at a significance level of *p* < 0.05.

(12) $$a^{*}=12.9-6.23X_{1}+0.831X_{2}-0.883X_{3}+0.1035X_{1}X_{1}-0.004600X_{2}X_{2}-0.00202X_{3}X_{3}+0.01815X_{1}X_{2}+0.05188X_{1}X_{3}+0.00222X_{2}X_{3}$$

The generation of the blend was significantly (*p* < 0.05) influenced by the interaction of feed moisture and barrel temperature, as well as the feed moisture and feed composition, with *p*-values of 0.001 and 0.000, respectively, at a significance level of *p* < 0.05. The influence of feed moisture, barrel temperature, and feed composition on the results, were significant (*p* < 0.05), which confirms our observation. The R^2^ and corrected R^2^ values were determined to be 94.94% and 93.63%, respectively. This indicates that the model equation could explain 94.94% and 93.63% of the variations, respectively. The coefficient of variation (CV) was calculated to be 2.14%, indicating a high level of precision in the sample data.

#### 3.1.5. Responses of the b* Color Index

All of the samples exhibited a positive b* index value, indicating a yellow hue. The b* index values represented in Table 3 varied from 24.85 to 37.95. These values were measured at a feed moisture level of 15 and 20, barrel temperatures of 100 °C and 115 °C, and feed compositions of 19% and 30%, respectively. The b* index value of the extrudate, which represents the degree of yellowness, exhibited an upward trend with higher barrel temperature, feed mix, and moisture levels [38]. The maximum value was observed within the temperature range of 115–130 °C, with a feed moisture level of 15–20 and pea integration of 19–30% (Table 3). This is believed to be due to the occurrence of browning reactions, such as the Maillard reaction due to the increased availability of simple sugars and amino acids necessary for Maillard Reaction in the malted flours, as well as the increased inclusion of pigeon pea.

**Figure 6:**
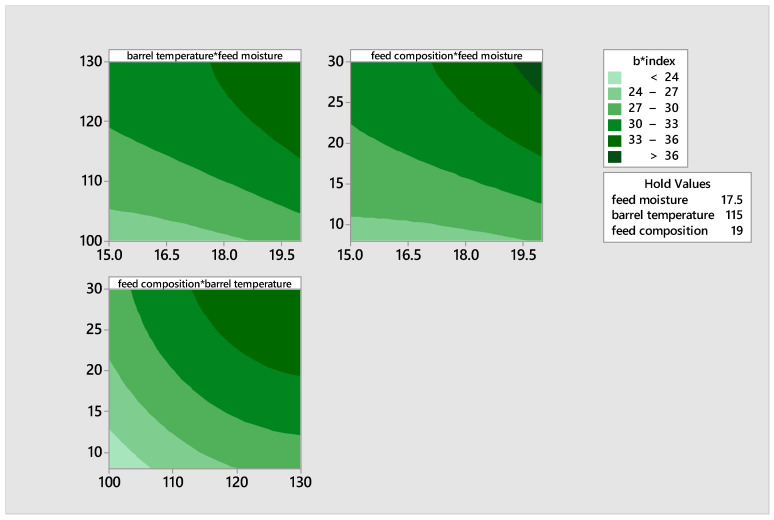
Contour plots illustrating the effects of moisture, feed composition, and temperature on the b* index of the product.

The b* index value of the extrudate exhibited a positive correlation with the barrel temperature, feed mix, and feed moisture content, as depicted in the contour plots (Figure 6). The values exceed the range of 13.13 to 28.80 reported by Danbaba et al. [38]. This is believed to be due to the magnitude of non-enzymatic browning reactions that occur when pigeon pea is added, as well as the caramelization of sugars from the malts that occurs as the temperature increases.

The quadratic model was statistically significant, with a *p*-value of 0.0000. Equation (13) displays the regression equation for the variable b* index.

(13) $$b^{*}=-25.7-4.32X_{1}+1.295X_{2}-0.306X_{3}+0.0750X_{1}X_{1}-0.00583X_{2}X_{2}-0.00881X_{3}X_{3}+0.01382X_{1}X_{2}+0.04700X_{1}X_{3}+0.00136X_{2\;}X_{3}$$

The linear factors, notably barrel temperature and its interaction with feed moisture and feed composition, had a significant impact on the developed blend at a significance level of *p* < 0.05. The quadratic effects of feed moisture and feed composition were similarly statistically significant, with *p*-values of 0.0000. The interactions between feed moisture and barrel temperature, as well as between feed moisture and feed composition, were found to be statistically significant. The *p*-values for these correlations were 0.048 and 0.0000, respectively, with a significance level of *p* < 0.05. The R^2^ and adjusted R^2^ values were determined to be 96.53% and 95.64%, respectively. The coefficient of variation (CV) was 1.6%, indicating a high level of precision in the samples.

#### 3.1.6. Changes in Color and Whiteness Index

The sample extruded at 20 feed moisture level, 115 °C barrel temperature, and 30% pigeon pea inclusion had the maximum color intensity for all color criteria. This is evidenced by the shift in color, with the highest significant value (50.99) observed under the same feed conditions (20, 115 °C, 30 %). The maximum whiteness index value of 75.75 was achieved at the center level for all parameters, including a feed moisture level of 17.5, a barrel temperature of 115 °C, and a feed composition of 19%.

The impact of malting on the color properties of the extrudates can be attributed to the rise in levels of simple sugars and amino acids during the malting process. This increase facilitates the Maillard reaction when the blends are subjected to high temperatures in the barrel, as well as the caramelization of sugars during heating. The Maillard reaction contributed significantly (*p* < 0.05) in determining the color of the extrudates. The color of samples with lower pea composition was lighter compared to samples with higher pea composition. This could be attributed to the increased presence of amino acids with increased pea addition and simple sugars from both flours, which support non-enzymatic browning. This indicates that the color of the grains is primarily a result of the Maillard and caramelization reaction from feed mix, with higher intensity observed at higher barrel temperatures.

### 3.2. Effects of Malting and Extrusion Variables on the Functional Properties of Rice–Pigeon Pea Extrudates

#### Bulk Density of the Extrudates

The extrudates’ bulk density varied between 0.72 and 0.84 g/cm^3^ under different conditions: feed moisture levels of 15 and 17.5, barrel temperatures of 130 °C and 100 °C, and feed compositions of 19% and 30%, respectively. Table 5 showed that extrudates with low intrinsic moisture content (7.40%) were less dense, whereas those with the highest bulk density had a higher moisture content of 8.10%. Aside from moisture, many factors such as un-gelatinized starch granules, the weight of the protein matrix, and fiber from the grains could have influenced the high bulk density. The extrudates in this study exhibited a higher bulk density compared to the value reported by Banki et al. [28], which varied from 0.05 to 0.11 g/mL. This phenomenon can be linked to the impact of malting and a significant quantity of fibers that can absorb moisture up to 30 times their own weight when hydrated.

**Figure 7:**
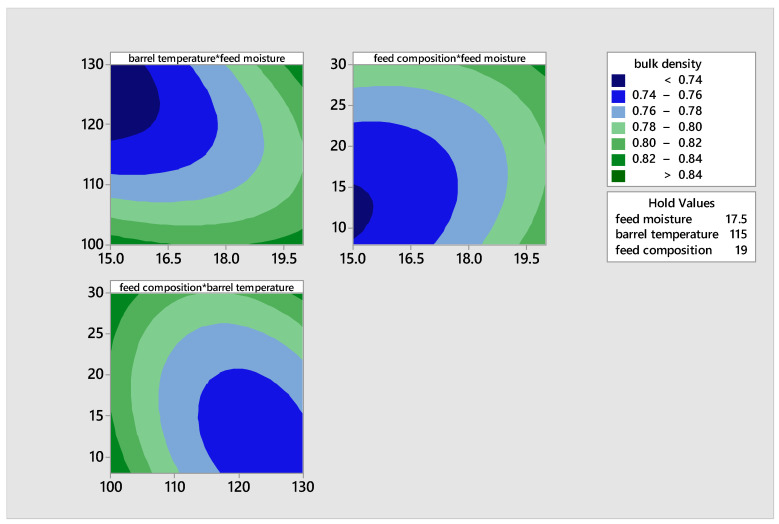
Contour plots illustrating the effects of feed moisture, feed composition, and barrel temperature on the bulk density of the product.

Figure 7 demonstrates that when the barrel temperature is high, feed composition is low, and feed moisture is low, the bulk density reaches its lowest value. Extrudates were less dense, with low feed composition and high barrel temperature. The high temperature likely decreased the minimum moisture content of the extrudates, resulting in reduced density, as moisture typically increases food density.

The increase in the bulk density of extrudates due to malting can be attributed to the high concentration of cellulose fibers from newly synthesized digested material. These fibers can absorb water that is 30 times their weight, resulting in an increase in bulk density when the feed composition is increased and hydrated. This effect is not observed in un-malted flours as reported by Banki et al. [28]

### 3.3. Swelling Capacity

**Figure 8:**
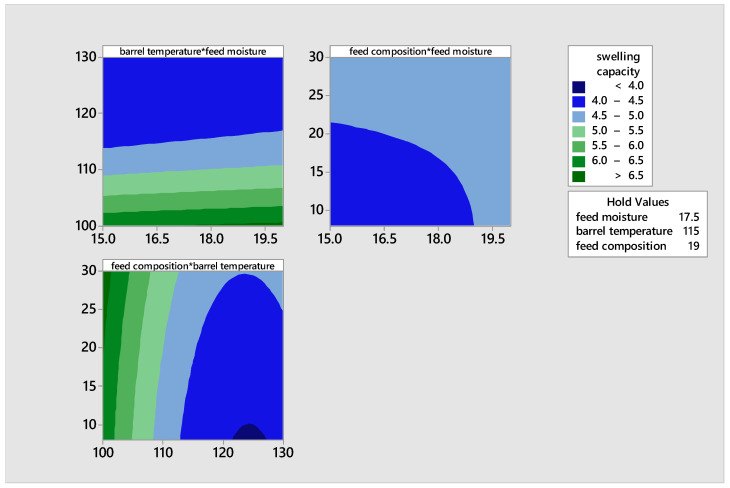
Contour plots illustrating the effects of moisture, feed composition, and temperature on the swelling capacity of the product.

The swelling capacity of the extrudates made from malted rice and pigeon pea flour blends varied from 3.95% to 6.50%, as shown in Table 5. The samples extruded at a feed moisture level of 17.5, a barrel temperature of 130 °C, and 8% pigeon pea composition exhibited the lowest swelling capacity. This may be attributed to the dextrinization process caused by the high temperature acting on the flour of malted grains, which primarily consist of sugars due to malting. The elevated temperature might have caused the flour mixture to undergo a change in viscosity, making it difficult for it to expand when in contact with water. The samples extruded at a feed moisture level of 20, a barrel temperature of 100 °C, and a pigeon pea composition of 19% exhibited the greatest swelling capability. Swelling capacity is a characteristic of starch, wherein the starch granules absorb water and become hydrated and swell. The low swelling capacity observed in samples extruded at high barrel temperature, regardless of the high feed moisture level, can be attributed to the property of heat to absorb moisture. This competes with the starch granules for moisture, thus limiting the swelling capacity and causing the viscous mass to have a melt viscosity. The swelling capacity contour plot graph (Figure 8) indicates a reduction in swelling capacity, regardless of the increase in pigeon pea content, when the barrel temperature is above 100 °C.

### 3.4. Water Absorption Capacity (WAC)

**Figure 9:**
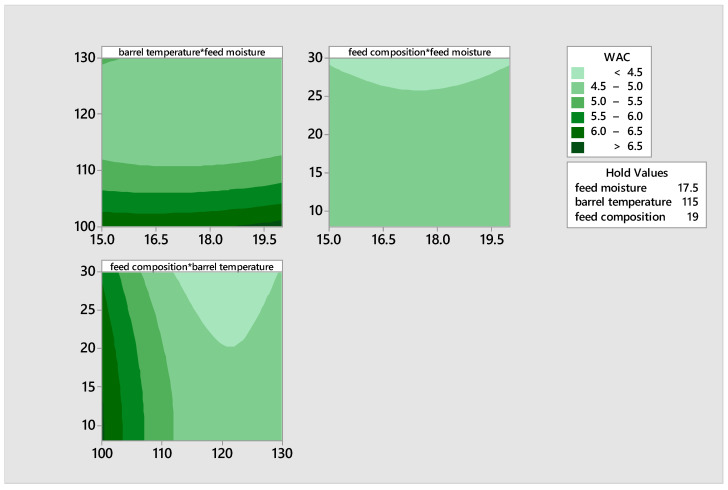
Contour plots illustrating the effects of moisture, feed composition, and temperature on the water absorption capacity of the product.

The water absorption capacity (WAC) quantifies the quantity of water that is primarily imbibed by starch and hydrophilic protein molecules. The water absorption capacity (WAC) of the extrudates ranged from 4.49% to 6.82%. The maximum WAC was recorded in the blend extruded with a feed moisture level of 20, a barrel temperature of 100 °C, and a pigeon pea content of 19%. The water absorption capacity decreased as the barrel temperature exceeded 100 °C. This can be attributed to the dextrinization of the starch granules which are converted to simple sugars during malting, resulting in a drop in the levels of WAC. Ding et al. [49] noted that the water absorption capacity (WAC) reduces as the temperature increases when dextrinization or starch melting are more significant (*p* < 0.05) than gelatinization. The observed drop in WAC as temperature increased is likely attributed to the decomposition or degradation of starch during malting and extrusion, even under conditions of high feed moisture and composition. The contour plot in Figure 9 confirms that the water absorption capacity (WAC) reduced as the temperature increases, particularly when dextrinization or starch melting are more significant than gelatinization.

### 3.5. Solubility

The solubility of the exudates ranged from 0.02 and 0.05, indicating a very small variation. The solubility of the extrudates increased as the barrel temperature and feed composition increased, with a drop in feed moisture. This phenomenon can be attributed to increased interaction between the large molecules of the mixture and heat in the presence of limited moisture. As a result, a greater number of these large molecules undergo degradation, transforming into more soluble forms, under high temperature and low moisture conditions. This process is commonly referred to as dextrinization. Dextrinization is the enzymatic breakdown of starch molecules into smaller, more soluble dextrin molecules, resulting in the browning of starch-based foods when exposed to high temperatures in a dry environment. Starch breakdown into dextrins, which are disaccharides, is the precise definition. The solubility of the extrudates is enhanced due to malting and the dextrinization process, which results in the creation of low molecular weight molecules during extrusion.

**Figure 10:**
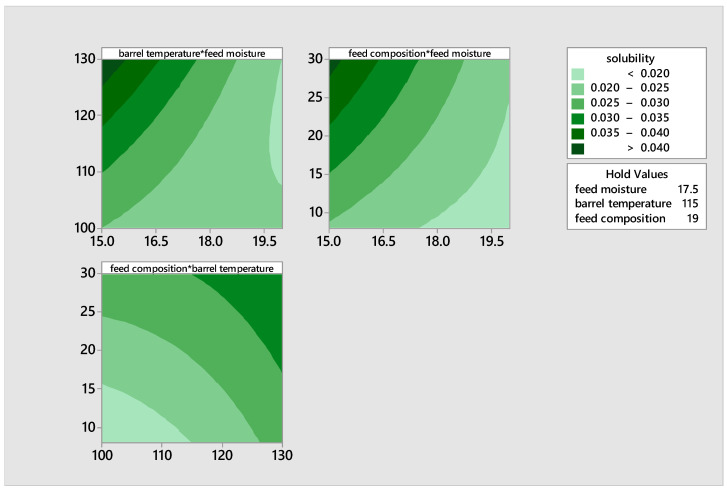
Contour plots illustrating the effects of moisture, feed composition, and temperature on the solubility of the product.

Figure 10 shows the contour plots illustrating the effects of feed moisture, feed composition, and temperature on the solubility of the product. There was an increase in the solubility of the extrudates, with an increase in barrel temperature and feed composition, and a decrease in feed moisture. The increase in solubility of the extrudates is as a result of dextrinization process that led to the formation of low molecular weight compounds during extrusion.

### 3.6. Viscosity

**Figure 11:**
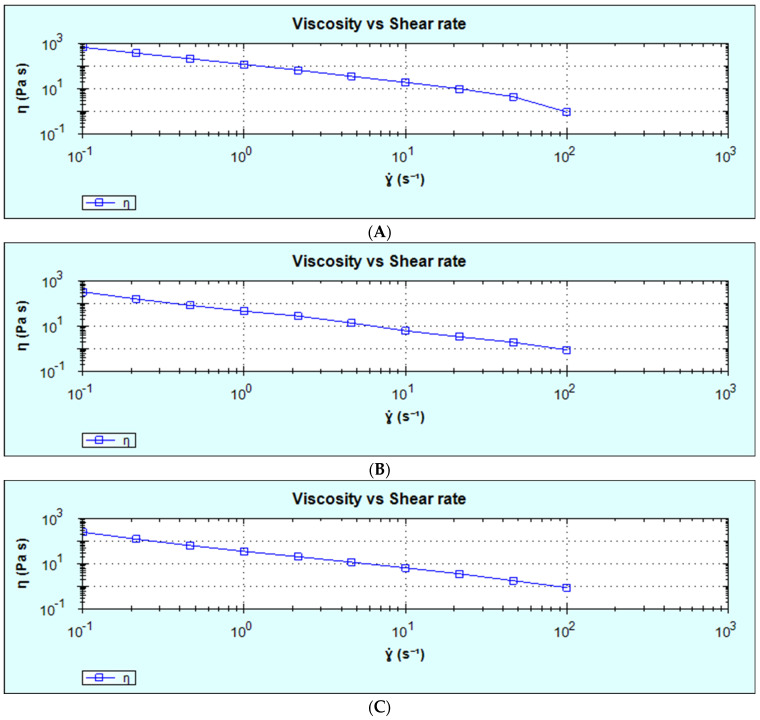
The viscosity and share rate of (**A**): sample 1 at varying design points (15, 100, 19); (**B**): sample 6 at varying design points (20, 115, 8); (**C**): sample 12 at varying design points (17.5, 130, 30), where (*X*_1_, *X*_2_, *X*_3_): (*X*_1_ = feed moisture, *X*_2_ = extruder barrel temperature, *X*_3_ = feed composition).

The viscosity property of all the samples showed a decrease in viscosity with increasing shear rate, as shown in Table 6. All the samples could be said to be a shear-thinning fluid (Figure 11A–C). This could be observed among all the samples, irrespective of the extrusion’s independent factors of feed moisture (15–20), barrel temperature (100–130 °C) and feed composition (8–30%). The reduction in viscosity among all the samples could be as a result of malting, which leads to the degradation of the macro molecules (starch and proteins) into simpler molecules (simple sugars and amino acids) leading to total melt viscosity when extrusion factors are applied [50]. According to Kristiawan et al. [51], starch transformation was assessed by the intrinsic viscosity, which was reflected in the extruded product’s molar mass. An increase in specific mechanical energy (SME) led to a drop in intrinsic viscosity between 20 and 80%, which was interpreted by starch depolymerization. The result was attributed to the properties of native starch, which resulted in a relative drop of intrinsic viscosity (%). According to Kristiawan et al. [51], at an SME interval of [250, 500 kJ/kg], the values of intrinsic viscosity rank inversely to their amylose content for maize starches. This is due to the larger molar mass of amylopectin, a highly short-branched macromolecule, which makes it more sensitive to shear at SME, than amylose, a linear macromolecule. The extent of intrinsic viscosity is the most important in the case of potato starch, from 60% to 80% at SME interval [250, 350 kJ/kg]. This trend can be explained by the higher value of intrinsic viscosity for its native starch (450 mL/g) compared to the values given for normal maize starch (amylose content of 23%) in the native state (22.0 mL/g), hence the larger size (molar mass) of potato starch macromolecules, amplified the sensitivity of this starch to shear.

### 3.7. Analysis of Variance of the Functional Properties of the Developed Quadratic Model for Germinated Brown Rice–Pigeon Pea Blend

The quadratic model for bulk density was found to be significant (*p* < 0.05), as shown in Table 7. Equation (14) represents the regression equation for the bulk density. The linear impact of the three parameters had a substantial influence on the bulk density of the extruded blends, with statistical significance (*p* < 0.05). The quadratic terms exhibited statistical significance with *p*-values < 0.05. The interaction between feed moisture and barrel temperature was found to be significant, as well as the interaction between barrel temperature and feed composition, with a significance level of *p* < 0.05. The R^2^ and adjusted R^2^ values were determined to be 88.98% and 84.02%, respectively, which indicated that 88.98% and 84.02% accounted for variation in the experiment. The lack of fit was not significant (*p* > 0.05), as indicated by a *p*-value of 0.639, which is desirable. The coefficient of variation was 1.09%, indicating the model’s dependability.

(14) $$\mathrm{Bulk}\;\mathrm{density}=4.949-0.1372X_{1}-0.05044X_{2}-0.01061X_{3}+0.002075X_{1}X_{1}+0.000148X_{2}X_{2}+0.000183X_{3}X_{3}-0.000723X_{1}X_{2}-0.000427X_{1}X_{3\;}-0.000110X_{2\;}X_{3}$$

The significance of the quadratic model for swelling capacity is shown in Table 7. Equation (15) represents the regression equation that describes the swelling capacity. The linear impact of the three components had a noteworthy influence on the swelling capacity of the extruded blends at a significance level of *p* < 0.05. The swelling capacity was shown to be positively correlated with the decrease in barrel temperature and the increase in pulse composition. The quadratic terms were statistically significant (*p* < 0.05). The interaction between feed moisture and barrel temperature, as well as the interaction between barrel temperature and feed composition, were found to be statistically significant (*p* < 0.05). The R^2^ and adjusted R^2^ values were determined to be 87.03% and 86.49%, respectively. This indicates that 87.03% and 86.49% of the variation can be accounted for by the model equation. The lack of fit was statistically significant, with a *p*-value of 0.00. The coefficient of variation was 8.47%, which signifies the experiment’s dependability

(15) $$\begin{array}{ll} \mathrm{Swelling}\;\mathrm{capacity} & =63.53+0.177X_{1}-1.0067X_{2}+0.0960X_{3}-0.00113X_{1}X_{1}\\ & \;\;\;\;\;+0.004013X_{2}X_{2}+0.000417X_{3}X_{3}+0.00033X_{1}X_{2}-0.00691X_{1}X_{3\;}\\ & \;\;\;\;\;+0.000273X_{2\;}X_{3} \end{array}$$

Table 7 displays the outcomes of the analysis of variance (ANOVA) for the quadratic models developed to measure water absorption capacity (WAC). Equation (16) displays the regression equation for the WAC expression. The linear, quadratic, and interaction effects of the three components significantly (*p* < 0.05) influenced the water absorption capacity of the blend, except for the interaction effect between feed moisture and feed composition. Equation (12) demonstrates that as each of the linear terms diminishes, there is an increase in WAC for the important linear factors. The R^2^ and adjusted R^2^ values were determined to be 88.98% and 84.02%, respectively. These values indicate that 88.98% and 84.02% of the variations, respectively, can be accounted for by the model equation. The lack of fit in the model demonstrating the reliability of the model was not significant (*p* > 0.05). The model demonstrated a high level of significance, as seen by the coefficient of variation (CV) of 1.09% in the WAC. This implies a reasonable amount of variation in the trials.

(16) $$\mathrm{Water}\;\mathrm{abs}\;\mathrm{capacity}=65.03-0.329X_{1}-0.9368X_{2}-0.0394X_{3}+0.0215X_{1}X_{1}+0.004076X_{2}X_{2}-0.001171X_{3}X_{3}-0.00367X_{1}X_{2}-0.00014X_{1}X_{3\;}+0.000568X_{2\;}X_{3}$$

Table 7 displays the outcome of the analysis of variance (ANOVA) conducted on the quadratic models designed for solubility. Equation (17) represents the regression equation that expresses the solubility of the extrudates. The combined effect of the three parameters had a negligible impact on the solubility of the extrudates at a significance level of *p* < 0.05. Furthermore, the barrel temperature did not exhibit any significant *p* > 0.05 impact on the linear and power effects. The R^2^ and adjusted R^2^ values were determined to be 86.55% and 80.50%, respectively, indicating that 86.55% and 80.50% of the variances, correspondingly, can be accounted for by the model equation. The lack of fit was statistically significant, although the model demonstrated a high level of significance with a coefficient of variation (CV) of 3.03% in solubility. This shows a reasonable amount of variation in the experimental results.

(17) $$\begin{array}{ll} \mathrm{Solubility} & =-0.165+0.0082X_{1}+0.00149X_{2}+0.00371X_{3}+0.000200X_{1}X_{1}\\ & \;\;\;\;\;+0.000006X_{2}X_{2}+0.000000X_{3}X_{3}-0.000133X_{1}X_{2}-0.000136X_{1}X_{3}\\ & \;\;\;\;\;-0.000008X_{2\;}X_{3} \end{array}$$

Table 8 shows the summary of the regression equation coefficients for the physical and functional variables of extrudates from flour blends of malted brown rice and pigeon pea.

### 3.8. Optimization of the Responses

By applying the desirability function, the best optimum independent variables for *X*_1_, *X*_2_, and *X*_3_ were 15, 130 °C, and 30 g/100 g, respectively (Table 9). At these combinations, optimum expansion index (5.00), b* (32.43), lightness (77.78), water absorption capacity (4.82%), water swelling capacity (4.73%), and solubility (0.05) were obtained, while minimum bulk density of 0.80 g/mL and moisture (6.57%) were obtained. The optimal desirability was 0.58, which indicates that about half of the responses were within the acceptability limits.

## 4. Conclusions

Malted pigeon pea had an effect on the physical properties of the extrudates in expansion index, bulk density, and color properties. Also, malted brown rice showed a significant effect on the physical properties of the extrudates, with samples containing a higher proportion of brown rice exhibiting higher expansion index and lightness on the extrudates. From the optimized functions, it was observed that optimum independent variables for feed moisture (*X*_1_), barrel temperature (*X*_2_), and feed composition (*X*_3_) were 15, 130 °C, and 30 g/100 g, respectively, in order to obtain the optimum expansion index, b* index, lightness, water absorption capacity, swelling capacity, solubility, and the minimum bulk density and moisture. It is recommended that flour blends of malted brown rice and pigeon pea should be extruded using the optimum independent variables, and its nutrient digestibility, availability, and overall sensory acceptability evaluated. This is because all developed foods must have an acceptable eating quality in order to contribute to achieving goal 2 of the sustainable development goals, ending hunger and achieving food security.

## Figures and Tables

**Table 1:** Outline of experimental design with coded and un-coded values.

Runs	Independent Variables in Coded Form			Independent Variables in Their Natural Form		
	*X*_1_	*X*_2_	*X*_3_	*X*_1_	*X*_2_	*X*_3_
1	−1	−1	0	15	100	19
2	1	−1	0	20	100	19
3	−1	1	0	15	130	19
4	1	1	0	20	130	19
5	−1	0	−1	15	115	8
6	1	0	−1	20	115	8
7	−1	0	1	15	115	30
8	1	0	1	20	115	30
9	0	−1	−1	17.5	100	8
10	0	1	−1	17.5	130	8
11	0	−1	1	17.5	100	30
12	0	1	1	17.5	130	30
13	0	0	0	17.5	115	19
14	0	0	0	17.5	115	19
15	0	0	0	17.5	115	19

*X*_1_ = feed moisture content, *X*_2_ = barrel temperature, *X*_3_ = feed composition; triplicate runs were carried out at all design points and the average was recorded. Run 13 was repeated two times.

**Table 2:** Feed composition mix (g) formulation design for each 1500 g sample/run.

Run	Germinated Brown Rice Flour (GBRF)	Non-Germinated Brown Rice Flour (BRF)	Germinated Pigeon Pea Flour (PPF)
1	546.75	668.25	285
2	546.75	668.25	285
3	546.75	668.25	285
4	546.75	668.25	285
5	621	759	120
6	621	759	120
7	472.5	577.5	450
8	472.5	577.5	450
9	621	759	120
10	621	759	120
11	472.5	577.5	450
12	472.5	577.5	450
13	546.75	668.25	285
14	546.75	668.25	285
15	546.75	668.25	285

GBRF: BRF (1:1.2); triplicate samples for each design point were prepared.

**Table 3:** Physical properties of extrudates from rice–pigeon pea flour blends.

Runs	Design Points (*X*_1_, *X*_2,_, *X*_3_)	Moisture Content (%)	Expansion Index	Lightness	a*	b*	Whiteness Index	Change in Color
1	(15, 100, 19)	8.79 ± 0.00	12.46 ± 0.71	73.74 ± 0.70	8.71 ± 0.25	24.85 ± 0.46	63.67 ± 9.59	34.47
2	20, 100, 19)	10.00 ± 0.00	12.33 ± 1.96	74.23 ± 1.34	9.45 ± 0.33	27.96 ± 0.99	63.72 ± 9.87	36.66
3	(15, 130, 19)	7.40 ± 0.28	5.17 ± 0.52	79.65 ± 0.07	11.24 ± 0.11	31.05 ± 0.19	63.90 ± 10.15	36.84
4	(20, 130, 19)	9.80 ± 0.00	3.92 ± 0.26	72.81 ± 0.62	14.70 ± 0.37	36.23 ± 0.53	64.08 ± 10.46	45.47
5	(15, 115, 8)	8.30 ± 0.13	12.94 ± 1.54	77.63 ± 0.45	9.00 ± 0.15	25.17 ± 0.34	64.91 ± 10.33	32.43
6	(20, 115, 8)	9.60 ± 0.00	7.26 ± 0.81	78.84 ± 0.45	8.70 ± 0.03	26.32 ± 0.16	64.90 ± 10.73	32.59
7	(15, 115, 30)	6.80 ± 0.85	5.56 ± 0.50	74.87 ± 0.40	12.08 ± 0.36	31.62 ± 0.71	64.88 ± 11.18	39.91
8	(20, 115, 30)	9.20 ± 0.28	3.5 ± 0.36	66.83 ± 2.84	17.48 ± 0.79	37.95 ± 0.72	65.52 ± 11.47	50.99
9	(17.5, 100, 8)	7.90 ± 0.14	12.06 ± 1.42	81.71 ± 0.53	6.95 ± 0.20	22.77 ± 0.48	67.40 ± 10.21	27.75
10	(17.5, 130, 8)	8.20 ± 0.84	5.24 ± 0.42	79.62 ± 0.20	10.28 ± 0.03	28.36 ± 0.51	67.12 ± 10.74	34.33
11	(17.5, 100, 30)	8.10 ± 0.14	8.71 ± 0.82	77.35 ± 0.84	9.25 ± 0.15	28.16 ± 0.35	67.56 ± 11.34	35.01
12	(17.5, 130, 30)	7.80 ± 0.28	4.24 ± 0.20	74.16 ± 0.44	14.05 ± 0.23	34.65 ± 0.51	68.26 ± 11.99	43.31
13	(17.5, 115, 19)	8.30 ± 0.14	4.85 ± 0.31	77.94 ± 0.29	11.39 ± 0.18	31.01 ± 0.25	70.54 ± 11.42	37.65
14	(17.5, 115, 19)	8.00 ± 0.28	4.98 ± 0.09	76.80 ± 0.96	11.29 ± 0.11	30.62 ± 0.15	72.59 ± 11.46	37.87
15	(17.5, 115, 19)	8.10 ± 0.14	4.96 ± 0.21	76.21 ± 0.72	11.55 ± 0.45	30.96 ± 0.87	75.75 ± 10.61	38.52
16	GBRF	12.29 ± 0.14		89.20 ± 0.38	2.31 ± 0.15	12.56 ± 0.35	81.25 ± 4.33	14.41
17	BRF	10.50 ± 0.42		91.11 ± 0.32	2.78 ± 0.12	11.89 ± 0.28	80.24 ± 5.14	13.11
18	PPF	6.70 ± 0.14		88.43 ± 1.72	4.60 ± 0.13	20.96 ± 0.94	75.58 ± 0.78	22.70

Key (***X*_1_**, ***X*_2_**, ***X*_3_**): (*X*_1_ = feed moisture, *X*_2_ = extruder barrel temperature, *X*_3_ = feed composition); GBRF = germinated brown rice flour; BRF = brown rice flour; PPF = germinated pigeon pea flour. Triplicate runs were carried out at all design points and the average was reported, except for lightness. a* = 0–60 for red and a− = 0–(−60) for green) b*: (b+ = 0–60) for yellow and b = 0–(−60) for blue.

**Table 4:** An analysis of variance of the physical properties of the developed quadratic model for the germinated rice–pigeon pea blend.

Source	Expansion Index *p*-Value	Moisture *p*-Value	Lightness *p*-Value	a* p-Value	b* p-Value
Model	0.000	0.000	0.000	0.000	0.000
*X*_1_ (feed moisture)	0.000	0.000	0.000	0.000	0.000
*X*_2_ (barrel temperature)	0.000	0.063	0.744	0.000	0.000
*X*_3_ (feed composition)	0.000	0.018	0.000	0.000	0.000
*X*_12_	0.000	0.000	0.000	0.000	0.000
*X*_22_	0.000	0.207	0.054	0.000	0.084
*X*_32_	0.000	0.039	0.466	0.235	0.000
*X*_1_ *X*_2_	0.092	0.051	0.000	0.001	0.048
*X*_1_ *X*_3_	0.000	0.071	0.000	0.000	0.000
*X*_2_ *X*_3_	0.000	0.312	0.527	0.068	0.380
Lack of fit	0.000	0.026	0.000	0.000	0.000
Coefficient of variation (%)	8.47	3.03	0.99	2.14	1.6
Coefficient of determination (R^2^)	87.03%	86.55%	86.53%	94.94%	96.53%
Adjusted R^2^	86.49%	80.50%	83.06%	93.63%	95.64%

Key (*X*_1_, *X*_2_, *X*_3_): (*X*_1_ = feed moisture, *X*_2_ = extruder barrel temperature, *X*_3_ = feed composition).

**Table 5:** The functional properties of the extrudates produced from rice–pigeon pea blends.

Runs	Design Points (*X*_1_, *X*_2_, *X*_3_)	Bulk Density	Swelling Cap (%)	WAC (%)	Solubility
1	(15, 100, 19)	0.83 ± 0.00	6.45 ± 0.07	6.64 ± 0.02	0.03 ± 0.01
2	20, 100, 19)	0.83 ± 0.00	6.50 ± 0.14	6.82 ± 0.10	0.02 ± 0.00
3	(15, 130, 19)	0.72 ± 0.00	4.30 ± 0.14	4.95 ± 0.08	0.05 ± 0.01
4	(20, 130, 19)	0.83 ± 0.00	4.40 ± 0.14	4.58 ± 0.01	0.02 ± 0.00
5	(15, 115, 8)	0.75 ± 0.00	4.05 ± 0.07	4.79 ± 0.01	0.02 ± 0.00
6	(20, 115, 8)	0.82 ± 0.00	4.80 ± 0.00	4.90 ± 0.05	0.02 ± 0.01
7	(15, 115, 30)	0.79 ± 0.00	4.70 ± 0.00	4.49 ± 0.05	0.05 ± 0.01
8	(20, 115, 30)	0.82 ± 0.02	4.69 ± 0.14	4.58 ± 0.04	0.03 ± 0.01
9	(17.5, 100, 8)	0.83 ± 0.00	6.28 ± 0.11	6.47 ± 0.26	0.02 ± 0.00
10	(17.5, 130, 8)	0.75 ± 0.06	3.95 ± 0.07	5.08 ± 0.06	0.03 ± 0.00
11	(17.5, 100, 30)	0.84 ± 0.01	6.90 ± 0.00	5.67 ± 0.17	0.03 ± 0.01
12	(17.5, 130, 30)	0.83 ± 0.00	4.75 ± 0.07	4.66 ± 0.04	0.03 ± 0.00
13	(17.5, 115, 19)	0.75 ± 0.03	4.58 ± 0.03	4.74 ± 0.06	0.02 ± 0.00
14	(17.5, 115, 19)	0.76 ± 0.01	4.38 ± 0.01	4.75 ± 0.24	0.03 ± 0.00
15	(17.5, 115, 19)	0.77 ± 0.00	4.59 ± 0.16	4.60 ± 0.11	0.03 ± 0.01

WAC = water absorption capacity. Key (*X*_1_, *X*_2_, *X*_3_): (*X*_1_ = feed moisture, *X*_2_ = extruder barrel temperature, *X*_3_ = feed composition). Triplicate runs were carried out at all design points and the average was reported.

**Table 6:** The rheology properties of sample 1 at varying design points (15, 100, 19), of sample 6 at varying design points (20, 115, 8), and of sample 12 at varying design points (17.5, 130, 30).

Sample	t (s)	Temperature °C	Force (N)	Share Stress (Pa)	Share Rate (s^−1^)	Viscosity (Pa·s)
Run1 at 25% Conc.	22.3	25.01	0.6186	71.47	0.1	714.7
	42.41	25.01	0.5862	83.41	0.2155	387.1
	62.52	25.01	0.5595	101.2	0.4642	218.1
	83.23	25.01	0.5215	123.1	1	123.1
	104.7	25.01	0.468	145.6	2.155	67.58
	125.8	25.01	0.4181	165.2	4.642	35.59
	146	25.01	0.3836	191.8	10	19.18
	208.3	25.01	0.2322	215.2	21.55	9.988
	245.5	25.01	0.1742	205.8	46.42	4.434
	364.8	25.02	0.09375	90.43	100	0.9043
Run 6 at 25% Conc.	100.8	25.01	0.8894	33.96	0.1	339.5
	125.4	25	0.677	35.01	0.2155	162.5
	145.8	25	0.5126	38.98	0.4642	83.99
	165.9	25	0.3856	46.43	1	46.43
	186	25	0.3179	60.73	2.155	28.19
	232.5	25	0.1235	64.31	4.642	13.85
	412.6	25	0.03763	62.28	10	6.227
	592.6	25.01	−0.07939	71.74	21.55	3.33
	612.7	25.01	−0.08936	88.3	46.42	1.902
	716.6	25.01	0.07892	87.46	100	0.8746
Run 12 at 25% Conc.	63.7	25	0.6088	25.81	0.1	258.1
	83.81	25	0.468	27.19	0.2155	126.2
	103.9	25.01	0.3483	30.21	0.4642	65.07
	124	25.01	0.2613	35.79	1	35.78
	144.1	25.01	0.2005	44.51	2.155	20.66
	168.9	25.01	0.1366	54.48	4.642	11.74
	199.1	25.01	0.07035	65.66	10	6.566
	219.2	25.01	0.03252	77.28	21.55	3.587
	239.3	25.01	2.17 × 10^−3^	82.51	46.42	1.778
	259.4	25	−0.03595	86.38	100	0.8637

Key (*X*_1_, *X*_2_, *X*_3_): (*X*_1_ = feed moisture, *X*_2_ = extruder barrel temperature, *X*_3_ = feed composition).

**Table 7:** An analysis of variance of the functional properties of the developed quadratic model for germinated brown rice–pigeon pea blend.

Source	Bulk Density (g/mL) *p*-Value	Swelling Cap *p*-Value	WAC *p*-Value	Solubility *p*-Value
Model	0.000	0.000	0.000	0.000
*X*_1_ (feed moisture)	0.000	0.000	0.000	0.000
*X*_2_ (barrel temperature)	0.000	0.000	0.000	0.063
*X*_3_ (feed composition)	0.000	0.000	0.000	0.018
*X*_12_	0.050	0.000	0.050	0.000
*X*_22_	0.000	0.000	0.000	0.207
*X*_32_	0.002	0.000	0.002	0.039
*X*_1_ *X*_2_	0.000	0.092	0.000	0.051
*X*_1_ *X*_3_	0.064	0.000	0.064	0.071
*X*_2_ *X*_3_	0.007	0.000	0.007	0.312
Lack of fit	0.639	0.000	0.639	0.026
Coefficient of variation (%)	1.09	8.47	1.09	3.03
Coefficient of determination (R^2^)	88.98%	87.03%	88.98%	86.55%
Adjusted R^2^	84.02%	86.49%	84.02%	80.50%

Key (*X*_1_, *X*_2_, *X*_3_): (*X*_1_ = feed moisture, *X*_2_ = extruder barrel temperature, *X*_3_ = feed composition).

**Table 8:** Estimated regression equation coefficients for the physical and functional variables of extrudates from flour blends of malted brown rice and pigeon pea.

Coefficient	Expansion Index	Lightness	a*	b*	Bulk Density	Swelling Cap	WAC	Solubility
Constant	239.8	−117.5	12.9	−25.7	4.949	63.53	65.03	−0.165
Linear								
*X*_1_	−9.44	22.07	−6.23	−4.32	−0.1372	0.177	−0.329	0.0082
*X*_2_	−2.095	−0.034	0.831	1.295	−0.05044	−1.0067	−0.9368	0.00149
*X*_3_	−1.396	1.281	−0.883	−0.306	−0.01061	0.0960	−0.0394	0.00371
Quadratic								
*X*_1_^2^	0.2634	−0.4432	0.1035	0.0750	0.002075	−0.00113	0.0215	0.000200
*X*_2_^2^	0.008406	0.00397	−0.004600	−0.00583	0.000148	0.004013	0.004076	0.000006
*X*_3_^2^	0.00614	0.00273	−0.00202	−0.00881	0.000183	0.000417	−0.001171	0.000000
Interaction								
*X*_12_	−0.00749	−0.0488	0.01815	0.01382	−0.000723	0.00033	0.00367	−0.000133
*X*_13_	0.03301	−0.0842	0.05188	0.04700	−0.000427	−0.00691	−0.00014	−0.000136
*X*_23_	0.000356	−0.00167	0.00222	0.00136	−0.000110	0.000273	0.000568	−0.000008
*R*^2^ (%)	87.03	86.53	94.94	96.5	88.98	87.03	88.98	86.55
*R*^2^_adj_ (%)	86.49	83.06	93.63	95.64	84.02	86.49	84.02	80.50

$Y=\beta_{0}+\beta_{1}X_{1}+\beta_{2}X_{2}+\beta_{3}X_{3}+\beta_{11}X_{1}^{2}+\beta_{22}X_{2}^{2}+\beta_{33}X_{3}^{2}+\beta_{12}X_{1}X_{2}+\beta_{13}X_{1}X_{3}+\beta_{23}X_{2}X_{3}\;$; Key (*X*_1_, *X*_2_, *X*_3_): (*X*_1_ = feed moisture, *X*_2_ = extruder barrel temperature, *X*_3_ = feed composition), expansion index, L* = lightness, a* = red to green, b* = blue to yellow, WAC = water absorption capacity (%), solubility index, bulk density (g/mL).

**Table 9:** Constraints and goals applied to derive optimum conditions of processing parameters and physical and functional responses for rice–pigeon pea based extrudates.

Variables	Goal	Lower Limit	Upper Limit	Importance	Optimum Level
**Independent Variables**					
Moisture content (% w.b)	In range	15	20	3	15
Barrel Temperature (°C)	In range	100	130	3	130
Feed composition (%)	In range	8	30	3	30
**Response Variables**					
***Physical properties***					
Bulk density	Minimum	0.72	0.84	3	0.80
Expansion index	Maximum	3.51	12.94	5	5.00
b*	Maximum	22.77	37.95	5	32.43
a*	Minimum	6.95	17.48	1	12.11
Lightness	Maximum	66.83	81.71	5	77.78
***Functional Properties***					
Water absorption capacity	Maximum	4.49	6.82	5	4.82
Swelling capacity	Maximum	3.95	6.90	5	4.73
Solubility	Maximum	0.01	0.04	5	0.05

## Data Availability

No research data were shared anywhere.
